# Integrating genome-wide association studies and transcriptomics prioritizes drug targets for meningioma

**DOI:** 10.1093/braincomms/fcaf053

**Published:** 2025-02-05

**Authors:** Wan-Zhe Liao, Jia-He Wang, Hua-Jie Zhong, Shen-Yu Wen, Yang Chen, Jia-Qi Chen, Xue-Kun Zhang, Xin-Yi Wu, Jia-Nuo Tan, Kun-Yi Li, Shao-Cong Mo, Li-Jun Wang

**Affiliations:** Nanshan College of Guangzhou Medical University, Guangzhou 511436, China; Nanshan College of Guangzhou Medical University, Guangzhou 511436, China; Nanshan College of Guangzhou Medical University, Guangzhou 511436, China; Nanshan College of Guangzhou Medical University, Guangzhou 511436, China; Nanshan College of Guangzhou Medical University, Guangzhou 511436, China; Nanshan College of Guangzhou Medical University, Guangzhou 511436, China; Nanshan College of Guangzhou Medical University, Guangzhou 511436, China; Nanshan College of Guangzhou Medical University, Guangzhou 511436, China; School of Stomatology, Guangzhou Medical University, Guangzhou 510182, China; The First Affiliated Hospital of Chengdu Medical College, Chengdu 610513, China; Department of Digestive Diseases, Huashan Hospital, Fudan University, Shanghai 200040, China; Department of Neurovascular Center, Changhai Hospital, Naval Medical University, Shanghai 200433, China

**Keywords:** meningioma, neuro-oncology, drug targets, single-cell sequencing

## Abstract

Meningioma, a prevalent central nervous system tumour, presents a significant challenge in neuro-oncology. This study harnesses genome-wide association studies (GWAS) and transcriptomic analysis to illuminate the pathological underpinnings of meningioma and spearhead the discovery of novel drug targets. By employing summary-data-based Mendelian randomization (SMR), colocalization analyses and Mendelian randomization, we pinpointed four genes as pivotal therapeutic targets. The integration of bulk and single-cell RNA sequencing confirmed the upregulated expression of three of the genes (*XBP1*, *TTC28* and *TRPC6*) in meningioma tissues, unravelling their cellular distribution and hinting at the tumour’s intrinsic heterogeneity. Molecular docking further identified dexamethasone and levonorgestrel as potential modulators of these targets, paving the way for personalized meningioma treatment strategies. This research advances our understanding of meningioma’s molecular landscape and illustrates the power of genomic and transcriptomic integration in the realm of precision oncology.

## Teaser

Liao *et al*. prioritized novel drug targets (*XBP1*, *TTC28* and *TRPC6*) for meningioma by integrating genome-wide association studies and transcriptomics, with RNA sequencing and molecular docking validating them and revealing expressive characteristics, advancing understanding of meningioma’s molecular landscape.

See Bertero, Padovan and Lombardi (https://doi.org/10.1093/braincomms/fcaf088) for a scientific commentary on this article.

## Graphical Abstract

**Graphical Abstract:**
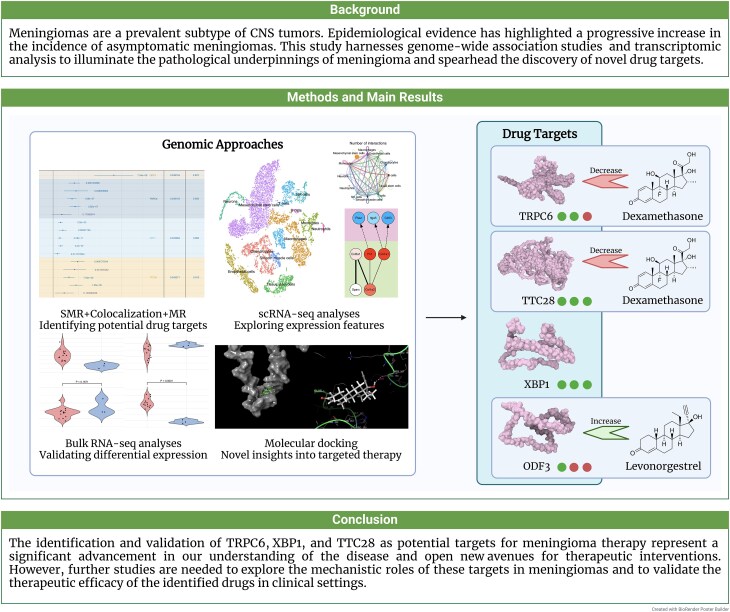

## Introduction

Meningiomas are a prevalent subtype of central nervous system tumours. Epidemiological evidence has highlighted a progressive increase in the incidence of asymptomatic meningiomas. Global epidemiological studies indicate the annual incidence rate of meningioma to be ∼8.83 per 100 000 individuals, with the likelihood of meningioma development increasing with age.^1^ Additionally, the overall 5-year survival rate of meningiomas exhibits age-dependent variations, with non-malignant cases ranging from 97 to 87.3%, and malignant meningiomas ranging from 85 to 50.2%. These findings highlight the significant challenges to global public health.^2^ The pathological features of meningiomas predominantly include the spherical proliferation of meningeal epithelial cells, resulting in the formation of whorl-shaped structures that ultimately mineralize into tumours. Characteristically, the chromatin within the tumour nucleus appears clear, with frequent invasion of the cytoplasm into the nucleus, giving rise to intranuclear cytoplasmic pseudoinclusions, which is a commonly observed phenomenon.^3^ According to the grading system established for central nervous system tumours, meningiomas are categorized into three grades: Grade I (benign), Grade II (atypical) and Grade III (anaplastic).^4^ Despite most meningiomas being benign and fully removable through surgery, other meningiomas pose a high risk of recurrence, especially WHO Grade II/III tumours (high-grade meningiomas), characterized by strong invasiveness and high postoperative recurrence rates, showing poor prognosis even with adjuvant radiotherapy,^4-6^ currently, no pharmacological treatment has been established for meningiomas.^7,8^ In contrast to benign meningiomas, malignant meningiomas are associated with a poor prognosis, frequently accompanied by local neurological deficits, seizures and a decline in quality of life.^9^ Therefore, acquiring a comprehensive understanding of the pathological mechanisms underlying meningiomas and actively investigating innovative therapeutic strategies is imperative.

**Figure 1:**
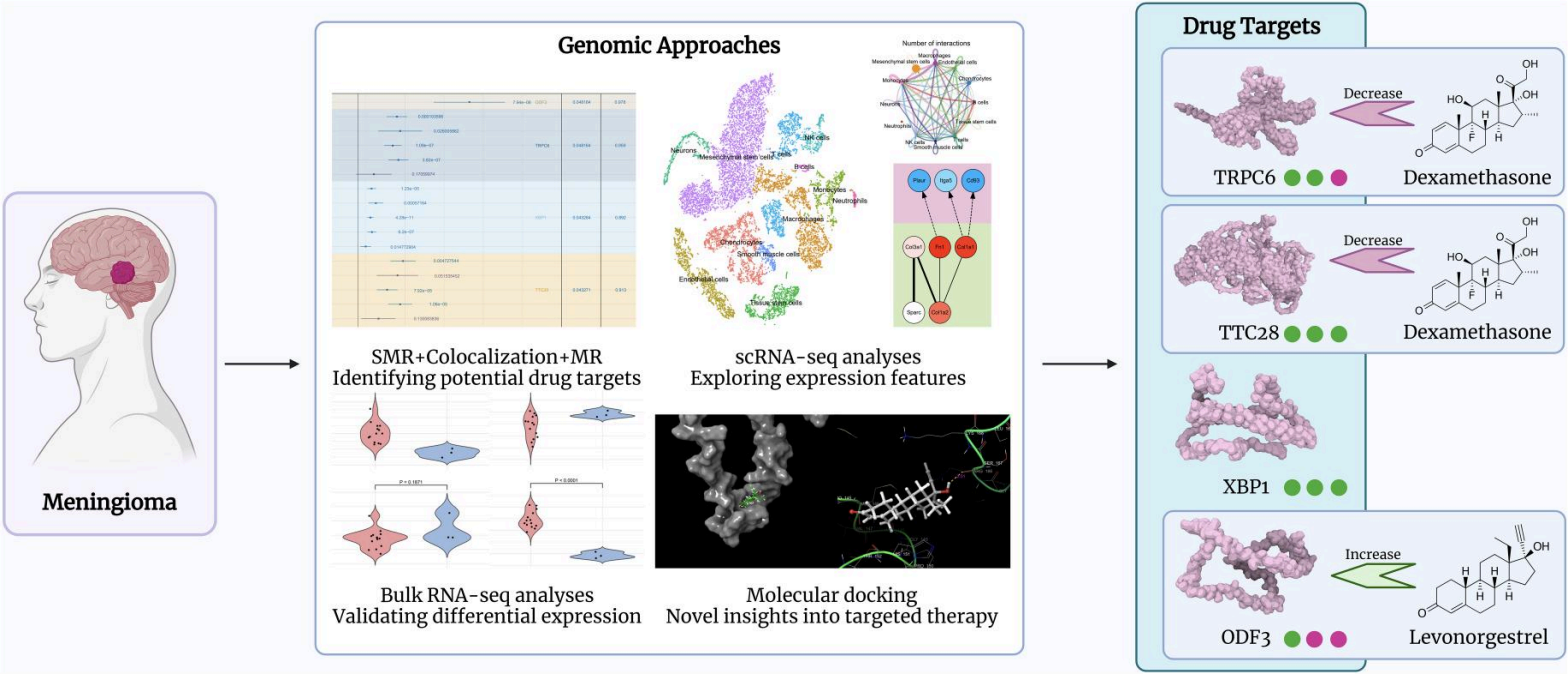
**Flowchart of the entire study.** We identified the causal genes for meningioma as potential drug targets by SMR, colocalization, and MR. Bulk RNA-seq and scRNA-seq analyses were conducted to validate their differential expression and expressive characteristics, and molecular docking was applied for novel insights into the existing medications for possible targeted therapy. The four drug targets are displayed on the right side, bound to the successfully docked drugs with their influences on the genes’ expression. SMR, summary-data-based Mendelian randomization; MR, Mendelian randomization; scRNA-seq, single-cell RNA-sequencing.

In recent years, several studies have used observational experimental approaches to investigate the causal relationship between meningiomas and high-dose progestins, including medroxyprogesterone acetate, potent progestogens and progesterone receptor expression. However, these research endeavours have several limitations, such as the omission of assessing the impact of cessation factors, subjectivity in the radiological diagnosis of meningiomas, and the lack of longitudinal data on untreated meningiomas.^10,11^ In this study, we diverged from conventional observational studies and used Mendelian randomization (MR), which leverages genetic variations as instrumental variables (IVs) for inherent randomization, to effectively mitigate the confounding factors and potential biases arising from reverse causality. This methodology facilitates the identification of potential drug target genes associated with meningiomas.^12^ Colocalization analysis techniques (COLOC) were employed to ascertain whether distinct gene expression signals converge within identical functional elements or regulatory regions, thus enabling more precise identification of potential drug targets.^13,14^ Moreover, to optimize the effectiveness of drug targets and elucidate the expression profiles of these target genes, we performed analyses involving bulk and single-cell transcriptomics. Unlike conventional bulk transcriptomics, single-cell transcriptomics enables the sequencing and analysis of RNA from individual cells, revealing variations in gene expression among distinct cells and cellular heterogeneity.^15,16^ Integrating both methodologies mutually enhances their utility, facilitating the analysis of potential targets while delineating cell type–specific targets and the feasibility of personalized therapy. In turn, this provides essential references and support for drug development and treatment strategies. Additionally, drug-protein docking was utilized to unveil the potential therapeutic effects of established drugs and to offer novel insights into targeted therapy for meningiomas. Overall, this study integrated genome-wide association studies (GWAS) and transcriptomics to prioritize drug targets and potential treatments for meningiomas. The whole analytical framework is shown in Fig. 1.

## Materials and methods

### Ethical approval

No additional ethics approval was needed because all the data in the study were previously collected, analysed and published.

### Data sources

Summary-level expression quantitative trait locus (eQTLs) data was retrieved from eQTLGen Phase I (https://eqtlgen.org/cis-eqtls.html), and GWAS summary-statistics for meningioma and the prioritized genes were stretched from FinnGen R9 (sample size = 287 614 (1237 cases versus 286 377 controls), released on May 2023, https://r9.finngen.fi) and ieu open gwas (https://gwas.mrcieu.ac.uk). Focusing on the transcriptomics, we obtained the GSE77259 (14 meningioma samples versus 3 normal brain tissue samples, bulk RNA sequencing) and GSE183655 [8 meningioma samples versus 2 dura samples, 57 114 cells, single-cell RNA sequencing (scRNA-seq)] from the Gene Expression Omnibus (GEO) online database (https://www.ncbi.nlm.nih.gov/geo) for downstream validation and exploration in brain tissues. No additional related ethical approval and informed consent was needed because all the data in the study were previously collected, analysed and published, and the sex of the analysed participants can be found in corresponding data sources (eQTLGen Phase I, FinnGen R9, ieu open gwas, GSE77529 and GSE183655 in GEO database).

### Summary-data-based MR analysis

We employed the sMR (SMR) software (version 1.03) to conduct SMR and the heterogeneity in dependent instruments (HEIDI) tests in the cis regions. Integrating summary data from GWAS with eQTL studies can enhance the ability to predict candidate genes for complex traits. The SMR analysis method is based on MR using a specific genetic variant at a prominent cis-eQTL as an IV.^17^ This was performed in combination with summary-level eQTLs data as the exposure and summary-level GWAS data for a specific trait as the outcome. This technique can reveal causal or pleiotropic relationships between gene expression and traits. In contrast, the HEIDI test can distinguish between causation and pleiotropy, as well as identify linkages [where two different single nucleotide polymorphisms (SNPs) in linkage disequilibrium independently affect gene expression and characteristics]. We processed the GWAS summary statistics for meningioma by removing low-frequency variants and SNPs that did not meet Hardy–Weinberg equilibrium criteria. Next, cis-eQTL data from the eQTLGen database were used, and SNPs within a 2 MB window around the gene probe were selected for analysis. Using the SMR command-line tool, we input the GWAS and eQTL data and set the significance threshold for the SMR test to 5.0 × 10^−8^ and the HEIDI test threshold to 0.01 to exclude false associations caused by linkage disequilibrium. For the HEIDI test, a *P*-value < 0.01 was considered significant, suggesting that the observed correlation was caused by linkage.

### Colocalization analysis

We performed a colocalization analysis using the coloc tool in R (version 4.3.2). The objective of COLOC analysis is to ascertain whether the SNPs linked to both gene expression and phenotype at a specific genomic location are causally related, hence indicating the ‘colocalization’ of gene expression and phenotype.^13^ To implement the analysis, we used the coloc.abf function, which computes posterior probabilities (PPs) for five hypotheses: (i) H0: no association with either trait; (ii) H1: association with gene expression only; (iii) H2: association with phenotype only; (iv) H3: association with both traits, but via different causal variants; and (v) H4: association with both traits via a shared causal variant. We considered strong evidence for colocalization when the PP for H4 exceeded 0.75, indicating common causal variants influencing both gene expression and phenotype.^13^ A Bayesian test was used to determine colocalization between pairs of genetic association studies by analysing the summary statistics. The default parameters of the coloc.abf function assigned a prior probability of 1 × 10^−4^ for H1 and H2, and a prior probability of 1 × 10^−5^ for H4. We tested the region spanning 1 MB on both sides of the lead mutation, which had the lowest *P*-value in the GWAS data.

### Mendelian randomization

In our MR investigating a subset of genes, the analysis workflow was as follows: (i) SNP selection, we selected genetic variants associated with gene expression at a genome-wide significance level from relevant eQTL data; (ii) data harmonization, we ensured that the effect alleles were aligned between the exposure and outcome datasets using the harmonize_data function; (iii) MR analysis, we applied all six methods using the mr function, specifying the method parameter accordingly (e.g. ‘mr_ivw’, ‘mr_egger’, ‘mr_weighted_median’, etc.); The MR methods employed were the Wald ratio, weighted mode, inverse variance weighted (IVW) mode, weighted median, MR-Egger and simple mode. The IVW method was chosen as our primary analysis due to its high statistical power when all IVs are valid. It provides a weighted average of the causal estimates from each genetic variant. The MR-Egger and weighted median methods were used to confirm the overall direction of exposure effects on the outcomes. These methods provide more reliable results even when the assumptions are not strictly satisfied, although they may have a slightly lower efficiency.^18,19^ Although several approaches have been used, *ODF3* consistently produced only one significant SNP as an IV in all selection processes. Therefore, we conducted a Wald ratio analysis exclusively for this gene.^20^

### Bulk RNA-sequence analyses

To evaluate the dependability of the target genes, we performed a search using the terms ‘Meningioma’ and ‘Homo sapiens’ and discovered the dataset GSE77259. This dataset comprised 14 meningioma samples and three normal brain tissue samples. We utilized the GEO2R web program to examine the unprocessed microarray data and detect genes that were differentially expressed in brain meningioma. Differential expression analysis was performed using the empirical Bayes method implemented in the package ‘limma’. To assess the diagnostic value of these genes for brain meningioma, we performed a receiver operating characteristic (ROC) curve analysis using the pROC R package (version 1.18.0). For each gene, we fitted a logistic regression model using the gene expression as the predictor and sample type (meningioma versus normal) as the outcome, then generated ROC curves by plotting the true positive rate against the false positive rate at various threshold settings. The area under the ROC curve (AUC), which is calculated using the trapezoidal rule, is a quantitative metric that combines the specificity and sensitivity of diagnostic tests. It indicates the internal effectiveness of these tests and ranges from 0.5 to 1. A large AUC value (>0.80) indicated superior diagnostic quality.^21^

### scRNA-seq analyses

scRNA-seq technology is the most advanced method for understanding the diversity and intricacy of RNA transcripts in individual cells. It also helps identify different cell types and their functions in well-structured tissues, organs, or organisms.^22^ To examine the variability between different genes’ expression in meningiomas, we obtained the GSE183655 dataset from the GEO online database. This dataset contains scRNA-seq data from a total of 57 114 cells derived from eight meningioma samples and two dura samples.

Single-cell transcriptomic technology was utilized in a range of procedures, such as data reanalysis, quality control, dimensionality reduction, annotation and differential analysis. Data reanalysis entails the first processing and refinement of raw data to remove any flaws or inconsistencies in sequencing, thereby guaranteeing an accurate and reliable interpretation of the outcomes. Quality control ensures the removal of low-quality cells and genes (related parameters were set as minGene = 500, maxGene = 20000, pctMT = 10 and pctHB = 3), thus ensuring the dependability and precision of subsequent analyses. Dimensionality reduction enables the representation of cell samples in two or three dimensions, facilitating identification potential cell subgroups. SingleR was used to annotate the cell clusters.^23^

We utilized a new computational method dubbed single-cell flux estimation analysis (scFEA) that allows for the assessment of the relative rate of metabolic flux at the level of individual cells using scRNA-seq data. The main assumptions of scFEA are that the flux changes of a metabolic module can be represented by a nonlinear function of the expression level changes of the enzymes involved and that the overall imbalance in flux of all intermediate substrates should be minimized in every single cell. We utilized scFEA on both the synthetic datasets and a newly created dataset with the corresponding scRNA-seq and metabolomic profiles. This validation revealed the ability of scFEA to predict accurately, withstand challenges and provide meaningful biological interpretations. The scFEA method allows estimation of the fluxome at the cellular level. This estimation enables identification of metabolic stress at the cellular or tissue level, evaluation of the sensitivity of particular enzymes to the overall metabolic network and inference of metabolic exchange between cells and tissues.^24^

CytoTalk is a computational technique that we developed to create cell type-specific signalling pathways using single-cell transcriptome data and known ligand-receptor interactions. CytoTalk fulfils the requirement of creating new and complete signalling pathways that are distinct for each cell type. Examination of these channels will improve our understanding of the intercellular communication between both normal and pathological tissues. The CytoTalk algorithm can be accessed using a software package on GitHub (https://github.com/tanlabcode/CytoTalk). Using CytoTalk, we performed a comparative investigation of the signalling pathways in human meningioma tissues. Our investigation revealed a greater diversity of signalling networks in normal tissues than those in meningioma tissues. Further, we discovered particular nodes in the network that displayed notable variations in signalling entropy across different tissues.^25^

Using the Cell-Chat technique, we identified the regulatory connections between cells that are associated with the characteristics of meningiomas in the cellular communication network. Interactions between cells were examined using the CellChat (v1.0.0) R program. CellChat offers a publicly available collection of ligands, receptors, cofactors and their interactions. This resource can be accessed at http://www.cellchat.org/. The Cell Chat R package is a flexible and intuitive set of tools for deducing, examining and illustrating cell-cell communication from any provided scRNA-seq data.

### Molecular docking

To examine small-molecule ligand medicines, we obtained their 2D structures from PubChem (https://www.rcsb.org/) and transformed them into 3D structures using the Chem3D software. Subsequently, we computed the least free energy. The 3D structures of the target proteins (receptors) were acquired from the RCSB Protein Data Bank (https://www.rcsb.org/) and subsequently enhanced using PyMOL (https://www.pymol.org/). We used the AutoDock Tool (version 1.5.6) to format the receptors and ligands as PDBQT files. We also created a 3D grid box to facilitate docking simulations of the receptors, with water and solvent removed. The main search algorithm was Lamarckian GA and cluster tolerance was set 2.00. Molecular docking was performed using the AutoDock4 software and meeko was chosen as ligand preparation tool. Binding sites with energies <−3 kcal/mol were deemed effective, and those below −5 kcal/mol were considered to have considerable binding activity. The most accurately predicted binding site was identified using PyMOL.^26^

### Statistical analysis

Statistical analyses were performed using R software (version 4.3.2). For the SMR analysis, *P*-values were corrected using the false discovery rate (FDR) method. In the HEIDI test, a *P*-value < 0.01 was considered significant for linkage causality. For colocalization analysis, a PP of at least 0.75 for H4 was considered strong evidence for shared causal variants. In the MR analysis, we primarily used the IVW estimate, with MR-Egger and weighted median methods used for additional confirmation. The AUC was calculated to assess diagnostic value, with AUC > 0.80 indicating good diagnostic quality. All statistical tests were two-sided, and *P*-values < 0.05 were considered statistically significant.

## Results

### SMR, COLOC and MR

**Figure 2:**
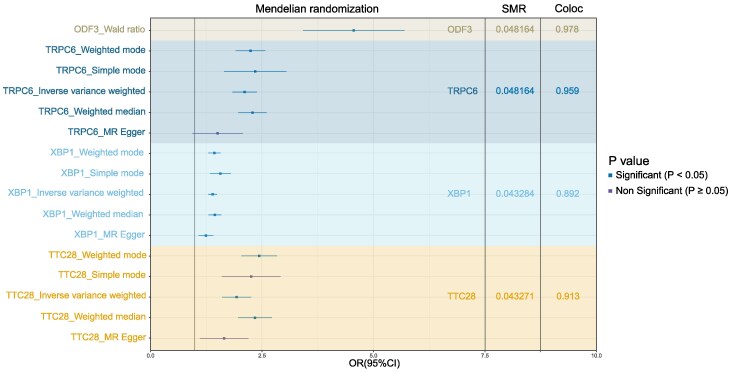
**Identification of potential drug targets by SMR, colocalization and MR.** Forest plot shows the MR results for the prioritized potential drug targets, with *P*-values attached. The numeric in the SMR column are the *P*-values of SMR results (corrected by FDR), the numeric in the Coloc column are the corresponding PP.H4. SMR, summary-data-based Mendelian randomization; MR, Mendelian randomization; OR, odds ratio; FDR, false discovery rate; PP.H4, posterior probability for hypothesis 4.

Firstly, we conducted SMR analysis and integrated GWAS and eQTL data to identify the most relevant genes significantly associated with meningioma traits. Four genes, *ODF3*, *TRPC6*, *XBP1* and *TTC28*, passed the SMR test (Fig. 2) after applying the FDR correction. We used COLOC to indicate a high likelihood of colocalization with the phenotype for *ODF3* (PP.H4 = 0.978), *TRPC6* (PP.H4 = 0.959), *TTC28* (PP.H4 = 0.913) and *XBP1* (PP.H4 = 0.892). We used MR to further validate their causality in meningioma. The results indicated that the expression of these four genes is causally stable in meningiomas (*TRPC6*: *P*^IVW^ = 1.09×10^−7^; *XBP1*: *P*^IVW^ = 4.28×10^−11^; *TTC28*: *P*^IVW^ = 7.92×10^−5^; *ODF3*: *P*^Wald ratio^ = 7.94×10^−8^). The odds ratios (ORs) of these genes were significantly high, indicating a positive causal relationship with meningiomas (*TRPC6*: OR^IVW^ = 2.11; *XBP1*: OR^IVW^ = 1.40; *TTC28*: OR^IVW^ = 1.93 and *ODF3*: OR^Wald ratio^ = 4.76).

### Bulk RNA validation

**Figure 3:**
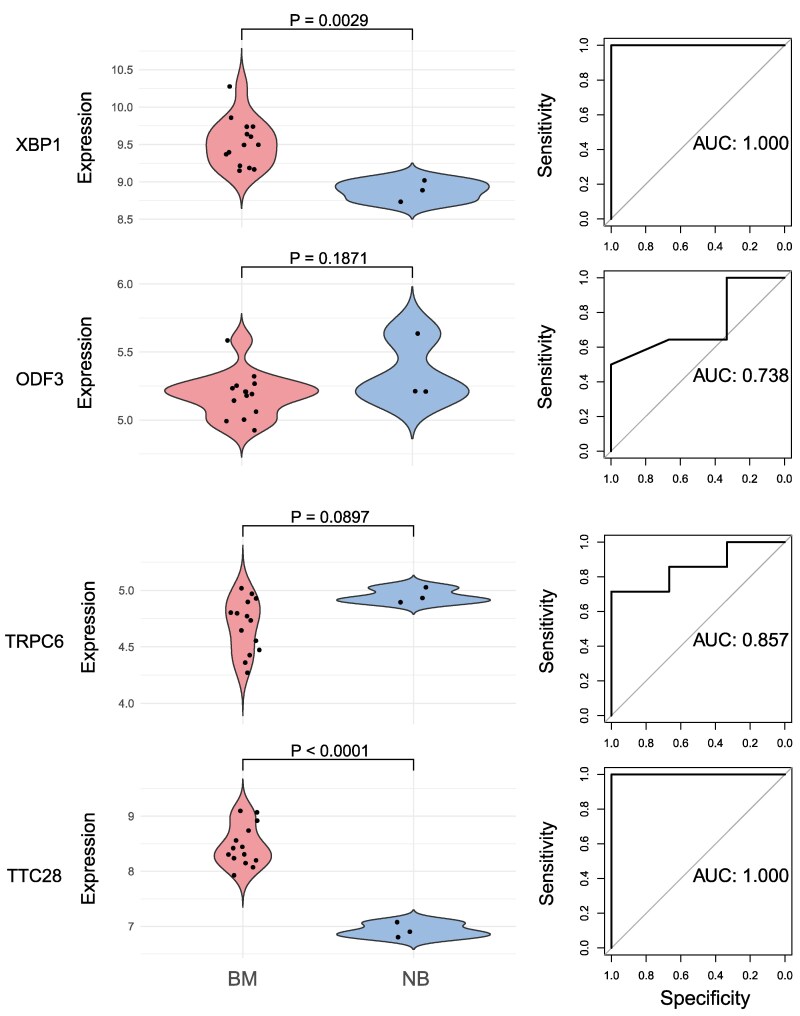
**Validation of the up-regulated expression and the diagnostic values of the identified genes in meningioma tissues by bulk transcriptome analysis.**  *P*-values are generated by Wilcoxon tests. BM, brain meningioma group; CT, control group.

Through bulk RNA validation, we successfully validated the specific expression of potential target genes screened previously. We focused on differences in the expression of the four identified genes (*XBP1*, *TTC28*, *TRPC6* and *ODF3*) between meningioma and normal brain tissues (Fig. 3). After conducting rigorous statistical analyses, the expression of *XBP1* and *TTC28*, was found to be significantly upregulated in meningioma tissues (*P* < 0.05), while *TRPC6* showed an increase with a *P*-value slightly beyond the commonly considered threshold of significance (*P* = 0.0897); however, *ODF3* showed no significant difference in expression. Furthermore, *XBP1*, *TTC28* and *TRPC6* showed relatively better diagnostic values in the ROC analyses (AUC > 0.80).

### scRNA analyses

**Figure 4:**
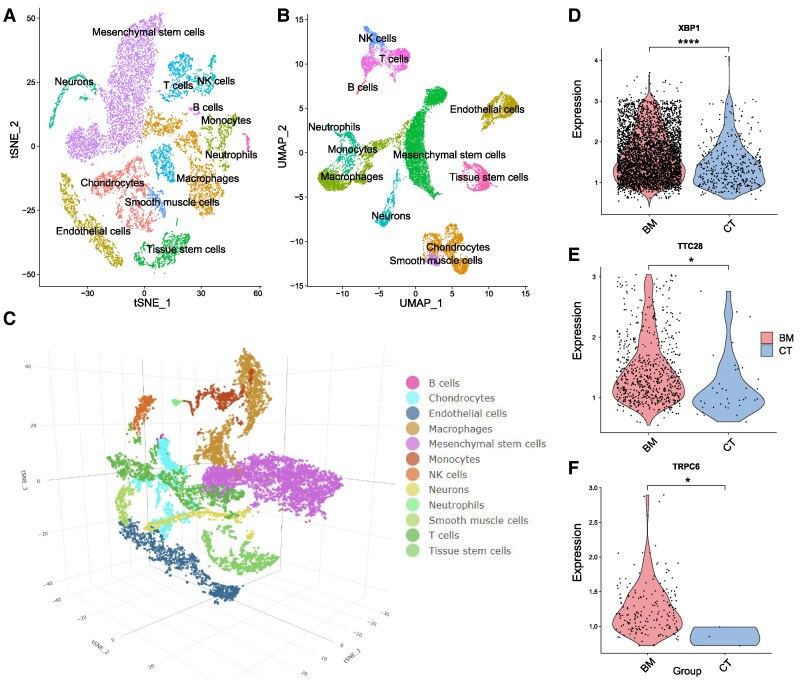
**Cell-type annotations and significant differences in the expression of the genes.** (**A** and **B**) The 2D tSNE and UMAP plots. (**C**) The 3D tSNE plot. (**D–F**) The expression difference between meningioma tissues and dura tissues (the control tissues) of *XBP1*, *TTC28* and *TRPC6*, using Students’ *t*-tests. *0.025 < *P*-value < 0.05; ****P-value < 0.001. BM, brain meningioma tissues; CT, control tissues; tSNE, t-distributed stochastic neighbour embedding; UMAP, uniform manifold approximation and projection.

To further validate potential drug targets and determine their expression characteristics in meningiomas, scRNA data were utilized. We applied two methods, t-distributed stochastic neighbour embedding (tSNE) and uniform manifold approximation and projection (UMAP), which effectively reduced the high-dimensional gene expression data to two or three dimensions. Cell division after downscaling is shown in Fig. 4A (downscaling by tSNE-2D), 4B (downscaling by UMAP-2D), and 3C (downscaling by tSNE-3D). By extracting specific cells in which the four prioritized genes were expressed, we validated the high expression of *XBP1*, *TTC28* and *TRPC6* in meningioma tissues (Fig. 4D–F).

**Figure 5:**
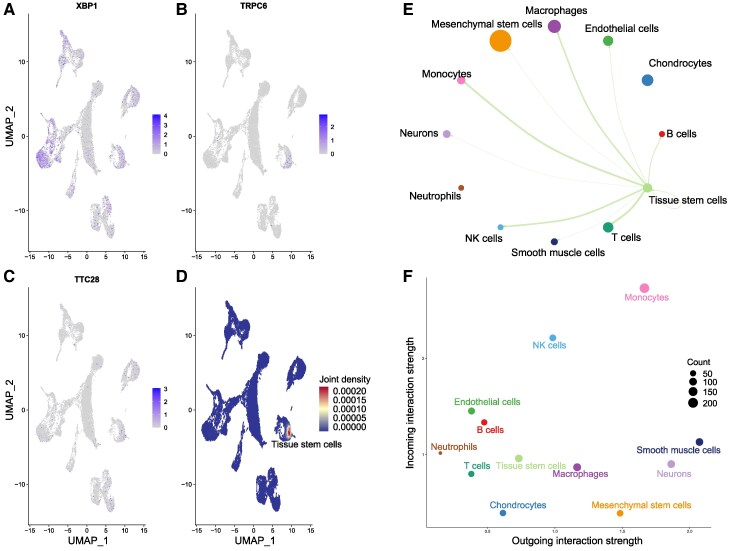
**Expression characteristics of *XBP1*, *TTC28* and *TRPC6*.** (**A–D**) The expression density of the three genes and identifying the cells that significantly co-express them. (**E**) The interactions among tissue stem cells and other cell clusters. (**F**) Incoming and outgoing interaction strength of the cell clusters. BM, brain meningioma tissues; CT, control tissues; UMAP, uniform manifold approximation and projection.

We analysed the three main genes associated with meningiomas and detected their expression in cells. *XBP1* showed the highest upregulation in macrophages from patients with meningioma compared with that in healthy individuals, with some upregulation in monocytes, tissue stem cells and NK (natural killer) cells (Fig. 5A). *TRPC6* was upregulated in tissue stem cells (Fig. 5B), whereas *TTC28* was relatively upregulated in the tissue stem cells, endothelial cells and chondrocytes (Fig. 5C). Joint density analysis revealed that *XBP1*, *TRPC6* and *TTC28* were significantly coexpressed in tissue stem cells (Fig. 5D). Using CellChat, we found that tissue stem cells had the most interactions with macrophages, monocytes, NK cells and T cells (Fig. 5E). The outgoing and incoming interaction strengths in each cell group were also determined (Fig. 5F). Using scFEA, we showed the characteristics of metabolic pathway abundance in cells expressing these genes, and revealed that Arginine to Ornithine, Leucine to Leucine, Ornithine to Putrescine, Orotidylic acid to UMP and Serine to Pyruvate were significantly enriched in *TRPC6*-, *XBP1*- and *TTC28*-expressing cells (Supplementary Fig. 1B). We also compared the proportion of cells expressing each gene and the gene expression levels in different cell populations between patients with meningioma and healthy individuals (Supplementary Fig. 1A). For example, *XBP1* was downregulated in B and NK cells from patients with meningioma compared with that in healthy individuals, whereas its expression level in macrophages and monocytes was upregulated in patients with meningioma.

**Figure 6:**
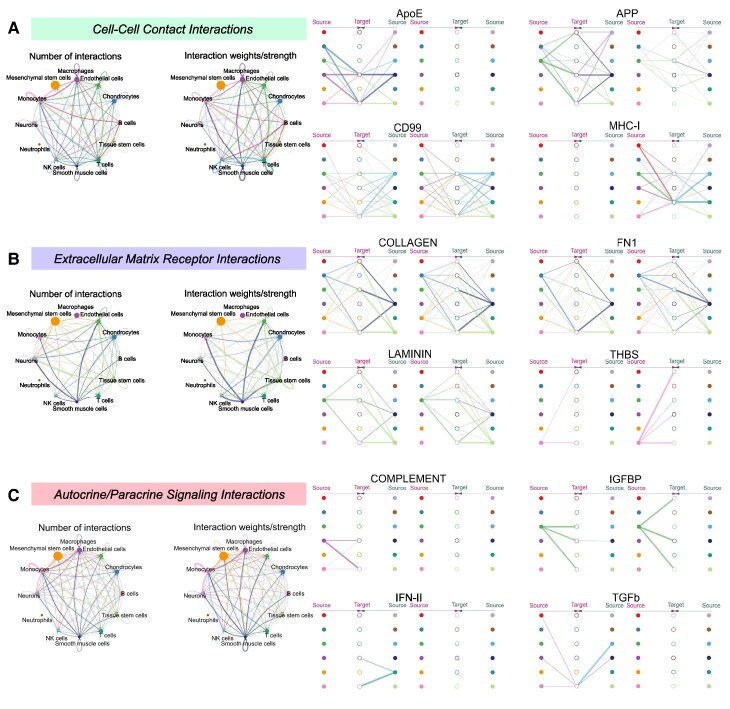
**Analysis of cell communication network related to tissue stem cells in meningioma.** (**A–C**) The secreted signalling interaction/cell–cell interaction/extracellular matrix receptor interaction network between cell clusters and the common ligand-receptor pairs in the corresponding interactions.

Concerning the tissue stem cells that co-express *XBP1*, *TRPC6* and *TTC28*, we analysed the cellular communication networks of tissue stem cells in different pathways using the data in CellChatDB (Fig. 6). Cell–cell interaction networks between cell clusters, extracellular matrix receptor interactions and autocrine/paracrine signalling interactions were generated. After comparing several signalling pathway networks, we discovered that tissue stem cells exhibited stronger outgoing and incoming interactions with other cell clusters in CD99, COLLAGEN and FN1 signalling pathway networks. Additionally, tissue stem cells in the LAMININ signalling pathway network exhibited strong outgoing interactions with other cell populations (Fig. 6A–C). Based on these findings, we employed CytoTalk to target the top four cell clusters with the highest intensity of interaction with tissue stem cells, namely macrophages, monocytes, NK cells and T cells, to investigate how these four clusters interact with tissue stem cells through ligand-receptor reactions. The analysis revealed that macrophages and NK cells interact with tissue stem cells through the Cd44/Col14a1 and Cd44/Col1a1 pathways. Monocytes interact with tissue stem cells through the Col1a1/Cd93, Col1a1/Itga5, Fn1/Plaur and Itgb1/Vcan pathways. In contrast, T cells interact with tissue stem cells via the Itgb1/Vcam1 pathway (Supplementary Fig. 2).

### Molecular docking

**Figure 7:**
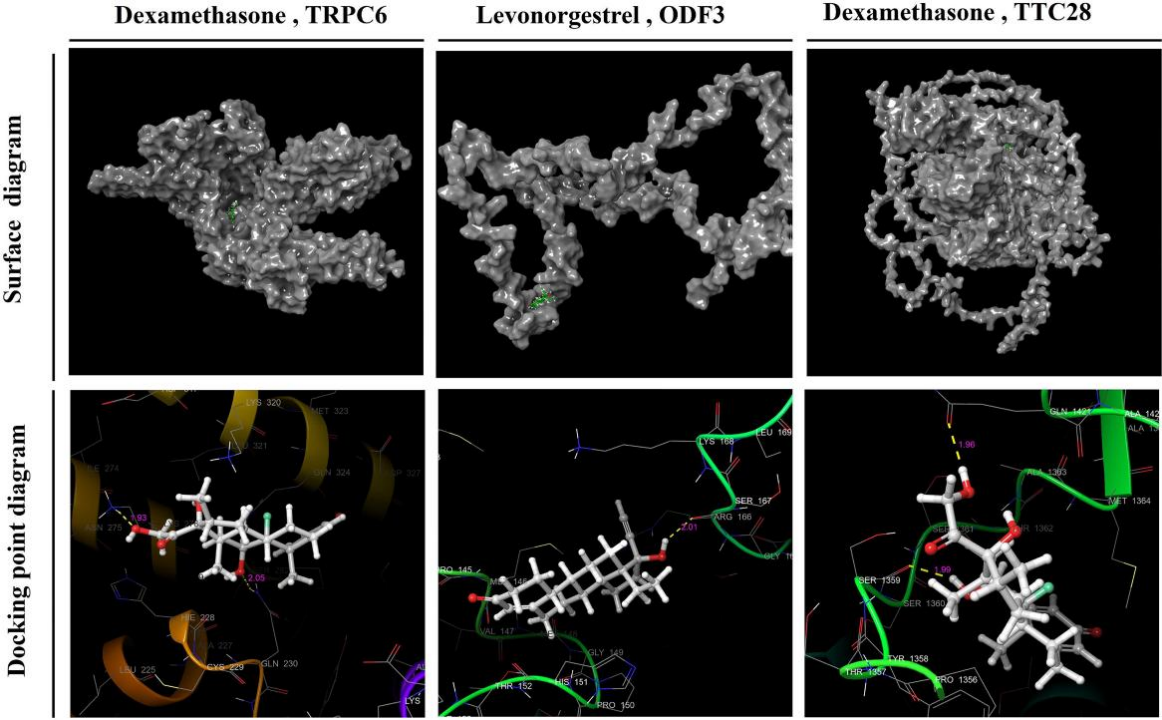
**Molecular docking diagram of potential therapeutic targets for meningioma and potential therapeutic drugs.** Binding energies: −3.302 kcal/mol for dexamethasone-*TRPC6*, −5.506 kcal/mol for dexamethasone-*TTC28* and −4.098 kcal/mol for levonorgestrel-*ODF3*.

Dexamethasone decreases the expression of *TRPC6* and *TTC28*. Levonorgestrel increases the expression of *ODF3*, lead chloride increases *XBP1* expression and lead acetate decreases *XBP1* expression. Among these, Dexamethasone-TRPC6, Dexamethasone-TTC28 and Levonorgestrel-ODF3 were successfully docked with effective binding activities (−3.302 kcal/mol for Dexamethasone-TRPC6, −5.506 kcal/mol for Dexamethasone-TTC28 and −4.098 kcal/mol for Levonorgestrel-ODF3; Fig. 7).

## Discussion

In this study, we performed SMR, colocalization and MR analyses using meningioma summary statistics from FinnGen r9 and eQTL data from eQTLGen, and identified *XBP1*, *TTC28*, *TRPC6* and *ODF3* as potential therapeutic gene targets for meningioma. We selected GSE77259, including 14 meningioma samples and three normal brain tissue samples. We also used GSE183655 scRNA-seq data of 57 114 cells from eight meningioma samples and two dura mater samples to perform scRNA-seq. *XBP1*, *TTC28* and *TRPC6* were stable during validation. Finally, We demonstrated the regulatory connections between cells associated with meningioma features in cellular communication networks using Cell-Chat.

We conducted a systematic analysis of potential drug targets for meningiomas using advanced analytical methods. Two gene prioritization methods have been established for post-GWAS analysis.^27^ The GIANT consortium performed a separate SMR analysis to combine its GWAS data for body mass index with 941 significant loci (*P* < 1 × 10^−8^) as well as eQTL data and prioritized 138 genes.^28^ Cordero *et al*.^29^ used both SMR and COLOC methods to identify three genes associated with COVID-19 hospitalization in lung tissue. However, our study extended this approach to meningiomas, illustrating the complex genetic landscape of the disease and identifying specific genes that may influence its progression and response to therapy. MR was used to screen *ODF3*, *TRPC6*, *XBP1* and *TTC28*. Research on drugs that directly target *ODF3* is still relatively scarce, and no effective drug has been found to downregulate *ODF3* expression to control the relevant disease development. According to the available studies, a relatively high percentage of *ODF3* gene expression may be associated with basal cell carcinoma^30^ and testicular cancer.^31^  *TRPC6* is a calcium ion channel that regulates the influx of calcium ions into cells, which is crucial for many signalling pathways, including those controlling cell proliferation, differentiation and apoptosis. The upregulation of *TRPC6* in meningioma, particularly in tissue stem cells, suggests its involvement in maintaining stem cell-like characteristics, which are often associated with tumorigenesis and resistance to conventional therapies. High *TRPC6* expression is mainly found glomerular diseases.^32^ Dysregulation of glomerular ion channels, such as changes in *TRPC6*, can lead to devastating glomerular disorders; many channels, including *TRPC6*, *TRPC5* and various ionotropic receptors, are promising targets for drug development, and our study demonstrates that the high expression of *TRPC6* in meningiomas may impact renal disease. Therefore, co-development of targeted therapeutic agents for *TRPC6* is important for meningiomas as well as renal disease. *XBP1* is a key regulator of the unfolded protein response (UPR), which is triggered by endoplasmic reticulum (ER) stress. In the context of meningioma, *XBP1*’s upregulation suggests that tumour cells may rely on the UPR to cope with increased protein synthesis and misfolding, conditions typical in rapidly growing tumours. *XBP1* expression is remarkably high in various disorders, especially in the context of an activated UPR;^33^ for example, in renal disease progression,^32^  *XBP1* activation is closely associated with the pathogenesis of kidney injury and with the transition from acute kidney injury to chronic kidney disease, which is marked by the up-regulation of fibrosis-associated genes and deposition of extracellular matrix. Chen *et al*.^34^ reported that *XBP1* expression was significantly higher in ovarian cancer than in normal ovarian tissues, and that its high expression significantly improved the overall survival and disease-free survival of patients with ovarian cancer. Our study is the first to identify the high expression of *XBP1* in meningioma, which may facilitate further exploration of meningioma-targeted drugs. Like *ODF3*, *TTC28* has been less well studied; *TTC28* is a lesser-known gene but has been linked to cell division and microtubule dynamics. In meningioma, *TTC28* is significantly upregulated in tissue stem cells, endothelial cells and chondrocytes. Its involvement in microtubule stabilization suggests that *TTC28* may contribute to tumour cell proliferation and chromosomal stability during cell division. This role is particularly important in rapidly dividing tumour cells, where precise microtubule dynamics are essential for accurate chromosome segregation. However, the atypical calcineurin Dachsous1b interacts with *TTC28* and Aurora B to control microtubule dynamics in embryo cleavage.^35^ Further, in a study by Chang *et al*.,^36^ significant expression of *XBP1* was detected in 20 protein-coding genes, 4 long-chain noncoding RNAs, and 10 non-translational regions, in which significant recurrent mutations were found. Functional analyses revealed that six genes with recurrent copy number variants in three squamous cell carcinomas (oesophageal, head and neck and lung) significantly promoted cancer cell proliferation, migration and invasion. The genes most frequently affected by the structural variants were LRP1B and *TTC28*. Therefore, further exploration of targeted agents for *TTC28* is necessary.

For in-depth profiling of scRNA-seq data, we employed two efficient downscaling methods, tSNE and UMAP, and by screening for cells expressing these genes and labelling them on downscaled plots, we found that these genes showed significantly higher expression in meningioma tissues than in dura tissues. Compared with healthy individuals, *XBP1* was the most upregulated in macrophages, and to some extent in monocytes, tissue stem cells, and NK cells. *XBP1* is a key transcription factor in the ER stress response and is essential for maintaining normal cellular functions and adaptive responses. Thus, *XBP1* upregulation may be attributed to its role as a key component of the ER stress pathway,^37^ which is activated to resolve protein folding errors and restore the stability of the intracellular environmen.^38^ Moreover, *XBP1* upregulation may affect immune cell function in the meningioma microenvironment, including the activation status of macrophages and NK cells, as well as their ability to recognize and clear tumour cells. By regulating the activity of these immune cells, *XBP1* may influence immune surveillance and the tumour therapeutic response and play a role in tumour immune escape. *TRPC6* is a calcium channel protein involved in regulating the intracellular calcium ion concentration, which affects cell function, suggesting that *TRPC6* participates in multiple signalling pathways by regulating intracellular calcium signalling, which maintains the undifferentiated state of stem cells and regulates their differentiation to specific cell lines. Calcium ions, as universal secondary messengers,^39^ play key roles in cell growth, differentiation and death. *TRPC6* upregulation in tissue stem cells may enhance the regulation of these processes. *TTC28* is upregulated in tissue stem cells, endothelial cells and chondrocytes. *TTC28*, a protein containing four tripeptide repeat domains, is commonly involved in protein-protein interactions involved in a variety of biological processes.^35^ This may be related to the meningioma microenvironment. In tissue stem cells, *TTC28* may regulate self-renewal and differentiation into different cell lines. In endothelial cells, *TTC28* upregulation may contribute to neoangiogenesis, which is essential for tumour growth and metastasis.

Using the data from CellChatDB, after comparing several signalling pathway networks, we found that tissue stem cells had stronger OUTGOING and INCOMING interaction strengths with other cell clusters in the CD99, COLLAGEN and FN1 signalling pathway networks. In addition to the above three, the LAMININ signalling pathway network of tissue stem cells has strong OUTGOING interactions with other cell populations. We thus suggest that CD99 is a cell-surface glycoprotein involved in various cellular processes, including cell adhesion, migration and immune cell activation. In tissue stem cells, the high communication activity of CD99 may be associated with cell migration and invasiveness in the tumour microenvironment, promoting tumour cell spread and metastasis, suggesting that *XBP1*, *TRPC6* and *TTC28* may promote tumour development. Collagen is a major component of the extracellular matrix and essential for maintaining tissue structure and function. The high interaction of tissue stem cells with collagen may affect the tumour microenvironment by promoting extracellular matrix synthesis and degradation, and influencing tumour growth and angiogenesis, suggesting that *XBP1*, *TRPC6* and *TTC28* may influence tumour growth and angiogenesis. FN1 is a glycoprotein involved in cellular adhesion, proliferation and migration. The role of FN1 in tissue stem cells with high communication intensity may facilitate interactions with other cells in the tumour microenvironment by supporting tumour cell attachment and spread, suggesting that *XBP1*, *TRPC6* and *TTC28* may influence the tumour microenvironment. LAMININ is an important extracellular matrix protein needed for cellular differentiation, migration and cell polarity maintenance. The strong outward communication of tissue stem cells in the LAMININ signalling pathway may indicate that they play a central role in the organization and functional regulation of the extracellular matrix, particularly in forming the tumour microenvironment and in the interaction of tumour cells with their surroundings, revealing a potential role for *XBP1*, *TRPC6* and *TTC28* in the extracellular matrix.

Using CytoTalk, the results showed that macrophages and NK cells interacted with tissue stem cells through the Cd44/Col14a1 and Cd44/Col1a1 pathways. Further, monocytes interact with tissue stem cells through the Col1a1/Cd93, Col1a1/Itga5, Fn1/Plaur and Itgb1/Vcan pathways. In contrast, T-cells interacted with tissue stem cells via the Itgb1/Vcam1 pathway. Interactions via the Cd44/Col14a1 and Cd44/Col1a1 pathways highlight the role of immune cells in monitoring the tumour microenvironment, which may be involved in modulating the inflammatory response and promoting tissue repair. Interactions with tissue stem cells through the Col1a1/Cd93, Col1a1/Itga5, Fn1/Plaur and Itgb1/Vcan pathways underscore the critical role of the monocyte-macrophage system in tumour immunosurveillance and immunomodulation. These pathways may influence cell adhesion, migration and the recognition and clearance of tumour cells by immune cells, further validating our results. Interaction with tissue stem cells through the Itgb1/Vcam1 pathway may reflect the complex role of T cells in regulating the meningioma tumour microenvironment, promoting immune responses and inhibiting or promoting tumour growth. The activation or inhibition of these pathways may provide targets for developing new therapies against the tumour microenvironment.

In meningiomas, we found that dexamethasone downregulates *TRPC6* and *TTC28* expression, as noted in the extant literature.^40^ Further, levonorgestrel downregulates *ODF3* expression. However, no well-docked drugs were identified for *XBP1*. These results reveal the effects of specific drugs such as dexamethasone and levonorgestrel on the expression of meningioma-associated genes, offering the possibility of targeting these genes for pharmacological intervention. Dexamethasone, a glucocorticoid, is known for its anti-inflammatory and immunosuppressive effects. It likely modulates *TRPC6* and *TTC28* by inhibiting pathways related to calcium signalling and cell division. In the case of *TRPC6*, dexamethasone has been shown to attenuate downstream signalling pathways, such as the calcineurin/NFAT axis, which is crucial for cell proliferation, migration and angiogenesis. By downregulating *TRPC6*, dexamethasone could potentially impair tumour growth by disrupting calcium-dependent signalling. Similarly, dexamethasone may modulate *TTC28* by influencing microtubule dynamics and cell division, thereby inhibiting tumour cell proliferation and angiogenesis. On the other hand, levonorgestrel, a synthetic progestin, modulates *ODF3* by interacting with progesterone receptors. Its ability to decrease *ODF3* expression suggests its potential to alter cellular structure and organization pathways, contributing to the inhibition of tumour cell growth and metastasis. Although levonorgestrel’s exact mechanism on *ODF3* remains to be fully elucidated, its impact on progesterone receptor pathways offers a promising therapeutic avenue for hormone-sensitive meningiomas. If future studies confirm an association of *TRPC6*, *TTC28* and *ODF3* expression levels with the clinical features and treatment response of meningiomas, monitoring the expression levels of these genes may help predict treatment responses to dexamethasone or levonorgestrel, thereby guiding the development of personalized treatment regimens. Although the effects of dexamethasone and levonorgestrel on *TRPC6*, *TTC28* and *ODF3* expression offer potential therapeutic strategies, no well-docked drugs have been identified for *XBP1*, indicating the need for further research.

Despite the significant findings for drug target exploration in meningiomas, our study still has limitations. Notably, our reliance on specific databases may have limited the comprehensiveness of the analysis. While these databases provide a wealth of data, they may not fully cover all relevant variables or the most recent findings. The size of the samples and their representativeness may have limited the generalizability of our findings. Further, methods like MR, SMR and colocalization analysis, although powerful, depend on specific statistical assumptions for validity. Although these studies are based on laboratory data and model analyses, they lack direct clinical trial validation. Therefore, clinical validation of the efficacy and safety of these drugs for meningioma treatment is required before translating these findings into therapeutic strategies. Although dexamethasone and levonorgestrel have the potential to be used in meningioma treatment, their possible side effects and long-term safety in clinical applications still need to be considered.

Future studies should thus focus on validating the therapeutic potential of *TRPC6*, *XBP1* and *TTC28*, both *in vitro* and *in vivo*. The therapeutic potential includes precise modification of DNA sequences using gene editing tools such as CRISPR-Cas9. By designing specific single guide RNAs to target a particular site within the target genes, Cas9 nuclease can be employed to induce site-specific cleavage, enabling gene knockout, replacement, or correction. Additionally, targeted therapies could intervene by focusing on the encoded proteins or their associated signalling pathways. Furthermore, exploring the mechanistic pathways by which these genes are involved in meningioma development and progression could help identify additional targets and inform the development of combination therapies.

In conclusion, the identification and validation of *TRPC6*, *XBP1* and *TTC28* as potential targets for meningioma therapy represent a significant advancement in our understanding of the disease and open new avenues for therapeutic interventions. However, further studies are needed to explore the mechanistic roles of these targets in meningiomas and to validate the therapeutic efficacy of the identified drugs in clinical settings. Overall, our findings contribute to the growing body of knowledge on meningiomas and represent a step forward in the development of effective treatments for this challenging disease.

## Supplementary Material

fcaf053_Supplementary_Data

## Data Availability

All the source data can be retrieved from the data sources described in ‘Methods’. The remaining part of the analytic codes are stored in https://github.com/Wanzhe-Liao/Integrating-GWAS-and-Transcriptomics-Prioritizes-Drug-Targets-for-Meningioma, should the readers contact the corresponding authors for details on other basic analyses if further concerns are raised.
