# Supplementary material for: Integrating genome-wide association studies and transcriptomics prioritizes drug targets for meningioma
Source: Brain Commun. 2025 Feb 5;7(2):fcaf053. doi: 10.1093/braincomms/fcaf053 (PMC11880806; doi:10.1093/braincomms/fcaf053)
Supplement: fcaf053_Supplementary_Data [file fcaf053_supplementary_data.zip › Supplementary Figure 2.pdf]

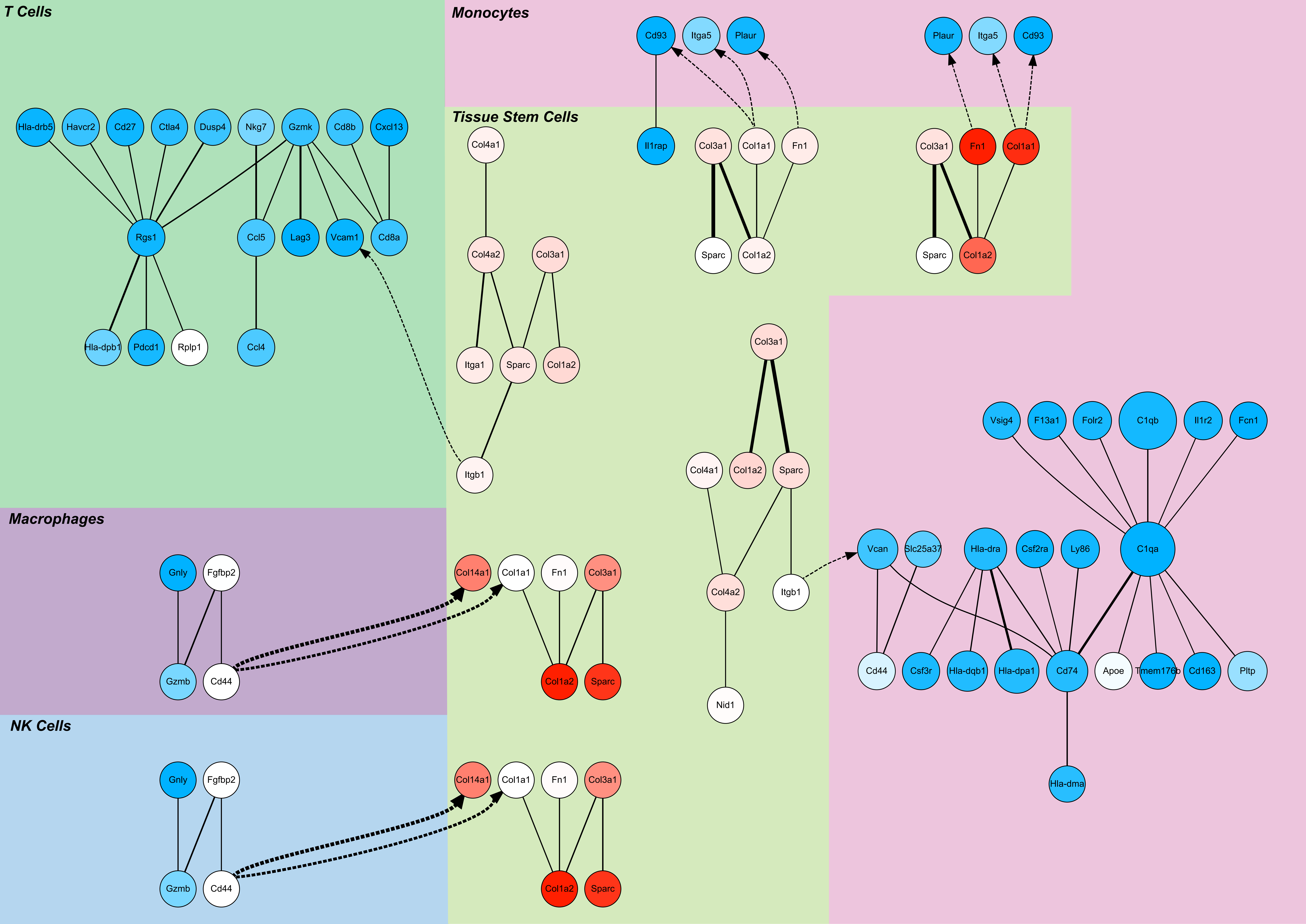
T Cells
Monocytes
Tissue Stem Cells
Macrophages
NK Cells
Hla-drb5
Havcr2
Cd27
Ctla4
Dusp4
Nkg7
Gzmk
Cd8b
Cxcl13
Rgs1
Ccl5
Lag3
Vcam1
Cd8a
Hla-dpb1
Pdcd1
Rplp1
Ccl4
Cd93
Itga5
Plaur
Il1rap
Col3a1
Col1a1
Fn1
Sparc
Col1a2
Col4a1
Col4a2
Itga1
Itgb1
Nid1
Vsig4
F13a1
Folr2
C1qb
Il1r2
Fcn1
C1qa
Vcan
Slc25a37
Hla-dra
Csf2ra
Ly86
Cd44
Csf3r
Hla-dqb1
Hla-dpa1
Cd74
Apoe
Tmem176b
Cd163
Pltp
Hla-dma
Gnly
Fgfbp2
Gzmb
Col14a1
