# Supplementary material for: Integrating genome-wide association studies and transcriptomics prioritizes drug targets for meningioma
Source: Brain Commun. 2025 Feb 5;7(2):fcaf053. doi: 10.1093/braincomms/fcaf053 (PMC11880806; doi:10.1093/braincomms/fcaf053)
Supplement: fcaf053_Supplementary_Data [file fcaf053_supplementary_data.zip › Supplementary_Figure_Legends.docx]

**Supplementary Figure 1**

**A.** The proportion of cells expressing the genes and the gene expression in different cell clusters between meningioma tissues and dura tissues. **B.** Metabolite abundance of the cells that express *TRPC6*, *XBP1*, or *TTC28*. BM, brain meningioma tissues; CT, control tissues.

**Supplementary Figure 2**

The pathways for the interaction of macrophages, monocytes, NK cells, and T cells with tissue stem cells. The intensity of each color indicates the preferential expression measure
