# Supplementary figures and images for: Integrating genome-wide association studies and transcriptomics prioritizes drug targets for meningioma
Source: Brain Commun. 2025 Feb 5;7(2):fcaf053. doi: 10.1093/braincomms/fcaf053 (PMC11880806; doi:10.1093/braincomms/fcaf053)

A

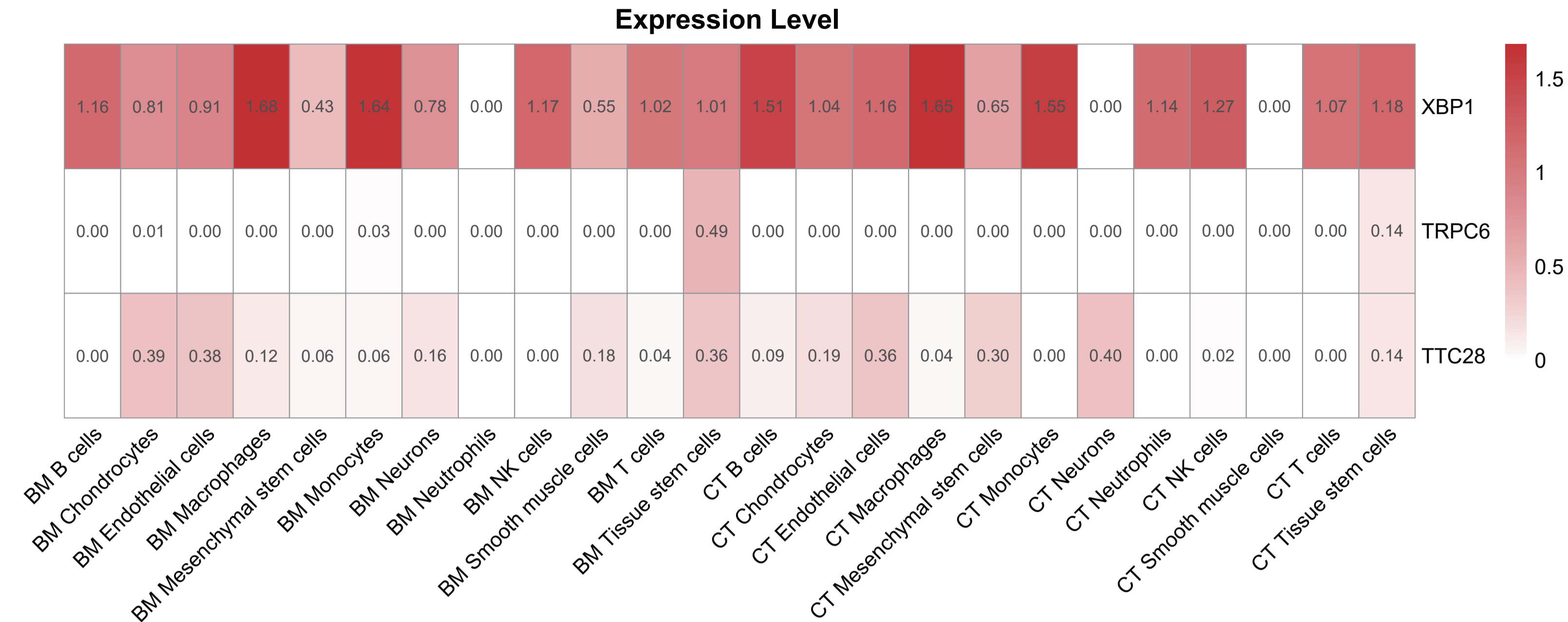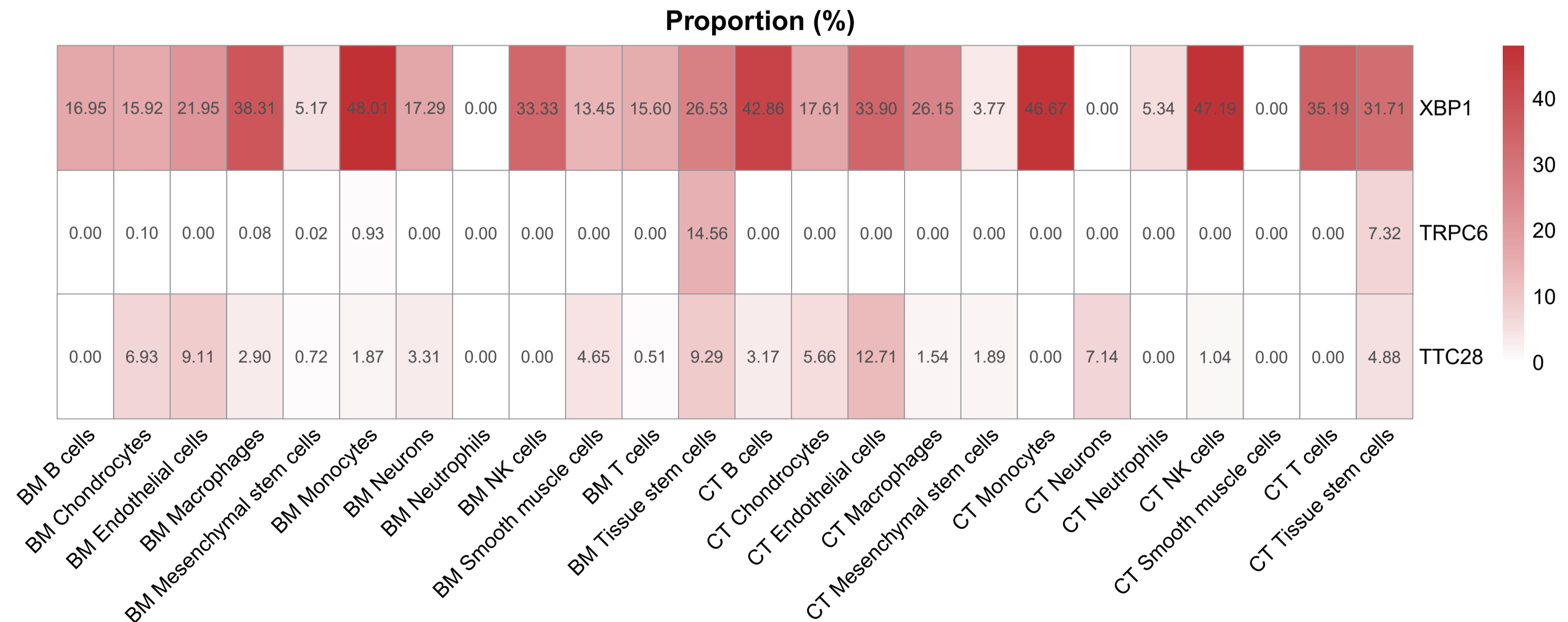

B

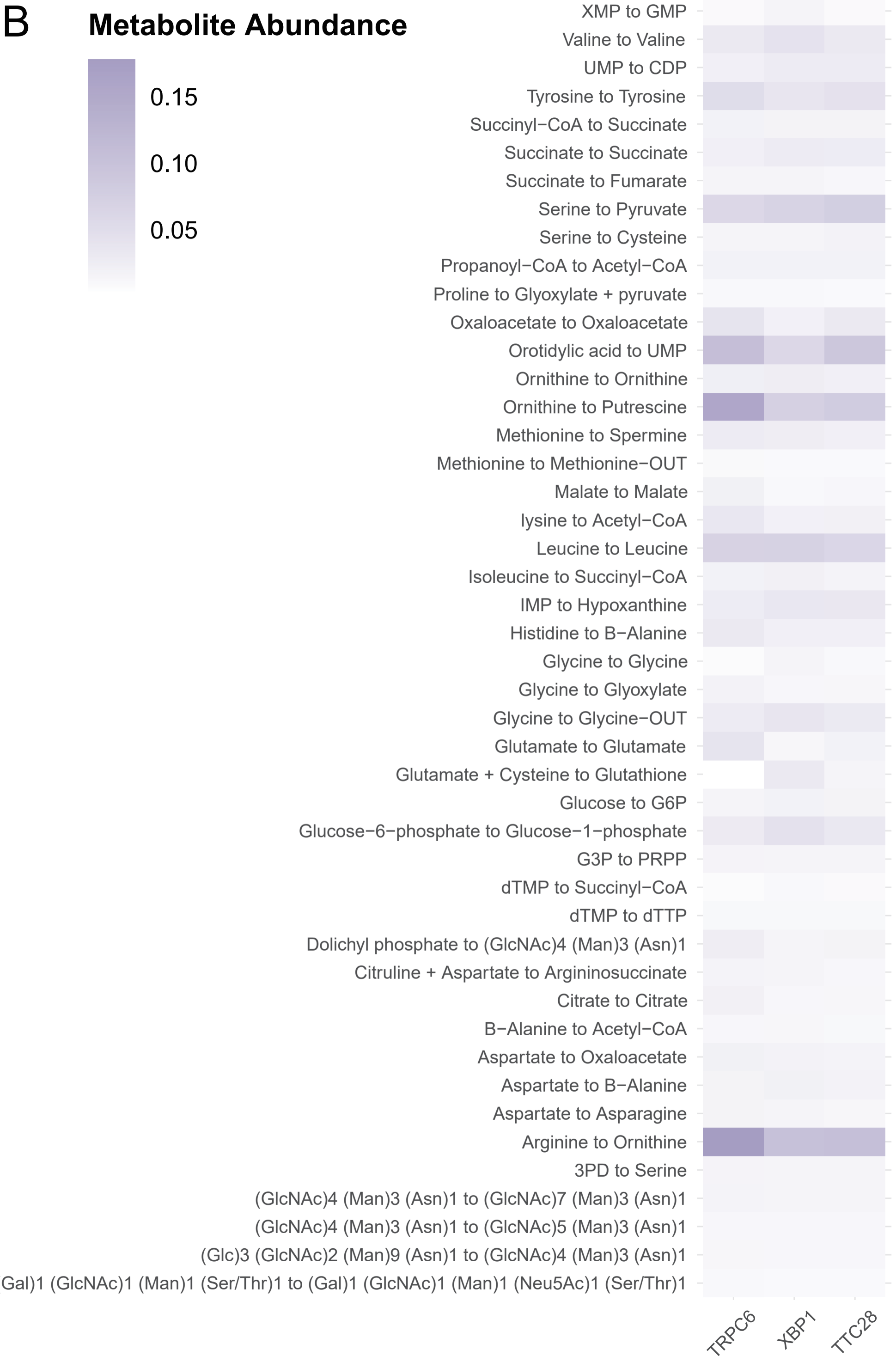

Supplement: fcaf053_Supplementary_Data [file fcaf053_supplementary_data.zip › Supplementary Figure 1.pdf]
